# Farnesol and Tyrosol: Secondary Metabolites with a Crucial *quorum-sensing* Role in *Candida* Biofilm Development

**DOI:** 10.3390/genes11040444

**Published:** 2020-04-18

**Authors:** Célia F. Rodrigues, Lucia Černáková

**Affiliations:** 1 LEPABE—Laboratory for Process Engineering, Environment, Biotechnology and Energy, Faculty of Engineering, University of Porto, Rua Dr. Roberto Frias, 4200-465 Porto, Portugal; c.fortunae@gmail.com; 2 Department of Microbiology and Virology, Faculty of Natural Sciences, Comenius University in Bratislava, Ilkovičova 6, 84215 Bratislava, Slovakia

**Keywords:** *quorum-sensing* molecules, farnesol, tyrosol, biofilm

## Abstract

When living in biological and interactive communities, microorganisms use *quorum-sensing* mechanisms for their communication. According to cell density, bacteria and fungi can produce signaling molecules (e.g., secondary metabolites), which participate, for example, in the regulation of gene expression and coordination of collective behavior in their natural niche. The existence of these secondary metabolites plays a main role in competence, colonization of host tissues and surfaces, morphogenesis, and biofilm development. Therefore, for the design of new antibacterials or antifungals and understanding on how these mechanisms occur, to inhibit the secretion of *quorum-sensing* (e.g., farnesol and tyrosol) molecules leading the progress of microbial infections seems to be an interesting option. In yeasts, farnesol has a main role in the morphological transition, inhibiting hyphae production in a concentration-dependent manner, while tyrosol has a contrary function, stimulating transition from spherical cells to germ tube form. It is beyond doubt that secretion of both molecules by fungi has not been fully described, but specific meaning for their existence has been found. This brief review summarizes the important function of these two compounds as signaling chemicals participating mainly in *Candida* morphogenesis and regulatory mechanisms.

## 1. Introduction

Biofilms are attached and structured microbial communities (single or polymicrobial), surrounded by an exopolymeric matrix. These entities are the predominant mode of microbial growth, offering several ecological advantages, such as nutrient availability, metabolic cooperation, protection from the environment, and acquisition of new traits. Most of them are particularly difficult to eradicate and are a source of many recalcitrant infections [1].

The higher density of microorganisms concentrated in one area and forming a biofilm requires communication between each other in a phenomenon called *quorum sensing* (QS) [2,3]. Indeed, a number of different types of secondary metabolites (SM) are released by fungi and bacteria. Typically, these secreted molecules have a low molecular weight and a variety of biologic tasks. While these compounds are not elementary to the central metabolism (e.g., growth and energy generation), SM are involved in biologic activities, which significantly help microbes surviving in an occupied ecological place [4].

**Figure 1:**
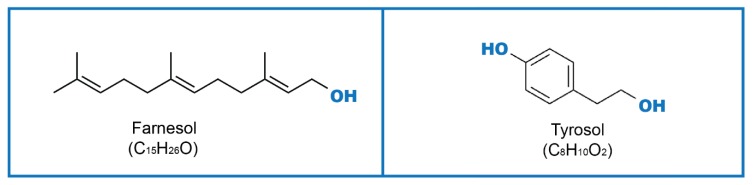
Farnesol (Far), Tyrosol (Tyr) molecular structures.

To achieve an effective cell–cell communication, microorganisms produce substances named *quorum-sensing* molecules (QSM), which control their response to external or internal stimuli. QSM such as farnesol (Far), tyrosol (Tyr) (Figure 1), phenylethanol, and tryptophol can be secreted by fungi, and their role has been investigated in both yeasts and filamentous fungi [5]. The effects of QSM are mainly described for morphogenesis (transition from spherical to hyphae form), initiation of fungal programmed cell death, apoptosis, and pathogenicity. In biofilm communities, QSM can affect biofilm (adhesion phase, proliferation, filamentation, maturation, and dispersion), regulation of cell morphology, and population density [5,6]. Products of microbial metabolism enable microorganisms to share information and, therefore, have an important signaling function in communication and control responses during both physiological and disease processes. These signaling molecules can be produced by fungi as well as by bacteria. Among fungi, QS mechanisms have also been described in filamentous fungi from the genera *Aspergillus* [7] and *Penicillium* [8,9]. In Gram-negative bacteria, signaling compounds are often acyl homoserine lactones, and in Gram-positive bacteria, they are usually modified peptides [10,11]. 

Metabolic profiling supports the identification of crucial determinant of pathogens and, hence, regulates infection progression [12]. QS are, in fact, a trade in cell signals that leads to a regulation of the fungal behaviors, depending on the density of the microbial population. Bacteria and fungi are under the control of these secreted QSM and impact morphogenesis, pathogenesis, biofilm formation, bioluminescence, and even the production of virulence factors. As a signaling mechanism, QS involves an exchange of low molecular weight chemicals called autoinducers. The accumulation of autoinducers in the extracellular space is also dependent on the increase of the community density. As so, gene expression or repression is controlled by autoinducers at a concentration level and QS allows single cells to react as multicellular organisms, determining their behavior, conducted by environmental cues [4,13].

Initially, QS was indicated as a particular system, exclusively of particular bacteria. The existence of QS systems in fungi was revealed only twenty years ago, after the discovery that Far manages filamentation in the pathogenic polymorphic yeast *Candida albicans* [3]. Its lead function in *C. albicans* physiology is linked to signaling and initiation of damaging consequences on host cells and other microbes [6,14]. After this discovery, the aromatic alcohol Tyr was also revealed to be a *C. albicans* QSM, managing growth, morphogenesis, and biofilm formation. Furthermore, in *Saccharomyces cerevisiae*, two other aromatic alcohols, phenylethanol and tryptophol, were found to be QSM participating in morphogenesis during nitrogen starvation conditions. As in bacteria and resembling QS, there is a population density-dependent behavior detected in several other fungal species. Even though fungal QS research is still in its beginning, the comprehension of fungal communication using signaling molecules showed us another potential approach in developing new therapeutics with antifungal effects [3,5,15].

Curiously, SMs are generally not essential to the existence of organisms’, since they can grow or reproduce without the presence of these compounds. Nevertheless, the release of SMs is an important process when adapting to an environment, or as a possible defense mechanism against predators, and thus, it helps in the survival of the microbial species [16]. Secondary products are secreted not only under common cultivation conditions and hence, the uncovering of chemical potential often requires the simulation of peculiar situations, in order to induce and awaken the associated biosynthetic genes. This is, for example, the case for change of growth media composition and cultivation conditions, which have been shown to effectively trigger the secondary metabolic pathways [17].

**Figure 2:**
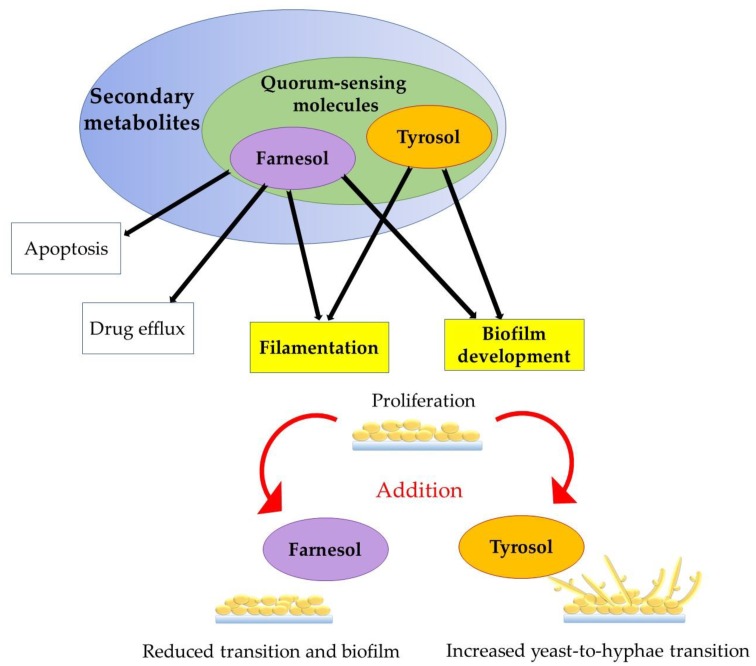
Role of externally added Far and Tyr to the proliferation stage as an important biofilm growth phase.

On the other side, QS inhibitors (QSI) have been shown to potentially be able to treat infections caused by bacteria, with the most perspective prokaryotes producing QSI likely to be those generally considered as safe. Among the eukaryotes, certain legumes and traditional herbs are also likely to work as QSI. Such findings are prone to lead to efficient therapy, lowering doses of commonly used antibiotics [18]. It is a fact that pathogens primarily control the expression of virulence genes using QS systems. QSI have been considered as promising antibiofilm compounds [19]. For example, *C. albicans* exhibit a complex QS system using these two SM with opposing effects and have been proposed to be consequential for biofilm processes [4,20]. Actually, an alternative antimicrobial treatment against fungi and bacteria with multidrug resistance phenomenon is now focused on targeting and inhibiting QSM, and also QSI [13]. Yet, much remains to be studied about the involvement of QS in biofilm development, management, and dispersion [19]. In this article, we give an overview of QSM, Far, and Tyr and their roles in *Candida* biofilm development (Figure 2).

## 2. Farnesol

Far (3,7,11-trimethyl-2,6,10-dodecatriene-1-ol; Figure 1) is an extracellular QSM, continuously produced in biofilms, during growth over a temperature range from 23 to 43 ℃, and in amounts roughly proportional to the colony-forming units per mL (CFU/mL). Chemically, Far is an acyclic sesquiterpene alcohol, endogenously synthesized via the ergosterol pathway, and it is a heat-stable molecule, unaffected by extreme pH (partly responsible for this protective reaction [21]). Far production is not dependent on the type of carbon nor nitrogen source, or on the chemical nature of the growth medium [20].

**Figure 3:**
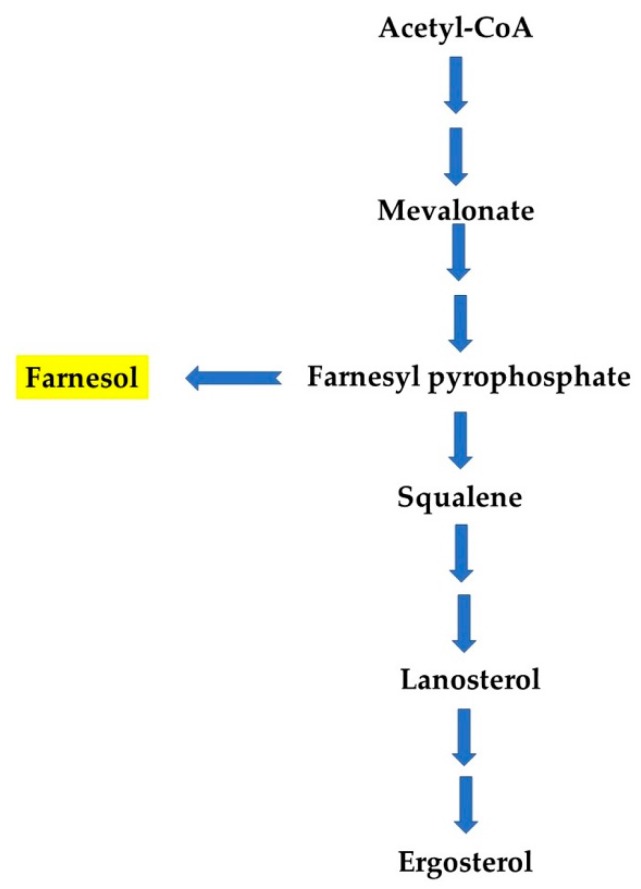
Far synthesis from a metabolic intermediate of ergosterol biosynthesis pathway, farnesyl pyrophosphate (according to References [22,23]).

Both natural and synthetic production of Far have different pathways in yeasts and bacteria. In yeasts, Far is a by-product from the ergosterol biosynthesis pathway, formed by enzymatic dephosphorylation of farnesyl pyrophosphate (Figure 3). Enzymes for this pathway are encoded by *ERG* (ergosterol) genes [22,23]. In bacteria, YisP (phytoene/squalene synthase), for example, acts as a phosphatase, catalyzing formation of Far from farnesyl diphosphate. Feng et al. described the role of YisP in *Bacillus subtilis* and showed that Far restored biofilm formation in a Δ*yisP* mutant, modifying the lipid membrane structure similarly to the virulence factor, staphyloxanthin [24]. Besides, Wang and colleagues described that farnesyl diphosphate accumulation can result in Far production in *Escherichia coli*. They found that PgpB (phosphatidate phosphatase) and YbjG (undecaprenyl-diphosphatase), two integral membrane phosphatases, can hydrolyze farnesyl diphosphate into Far and construct a novel Far synthesis pathway for mass production in *E. coli* [25]. A large-scale production of Far can also be achieved using chemical synthesis and metabolic engineering approaches. Importantly, Far and its derivatives/analogues have been reported to exhibit anti-biofilm, anti-cancer, anti-tumor, and fungicidal properties. It is important to mention that the anti-biofilm activity of Far has been described according to time of administration, as well as used concentration to inhibit *Candida* biofilm development, which is explained in the following paragraphs. Yet, the impact of Far on bacterial biofilm is less explored. The antimicrobial potential of Far has been enhanced by synergizing it with known antifungal drugs, and through nano-formulation(s). Therefore, apart from its QS activity, Far can be used as an effective anti-microbial, anti-inflammatory, anti-allergic, and anti-obesity agent [14], and several studies have also revealed that Far affects the growth of several bacteria and fungi, pointing to a potential role as an antimicrobial agent [26].

As acknowledged, Far is involved in the inhibition of hypha formation, regulation of various physiological processes including filamentation, biofilm formation, drug efflux, and apoptosis [6,14,27,28]. This compound is produced by many organisms, mainly *Candida*, and also found in several essential oils [26,29]. The secretion of Far was confirmed under various conditions in eight *Candida*: *C. albicans*, *Candida dubliniensis*, *Candida tropicalis*, *Candida parapsilosis*, *Candida guilliermondii*, *Candida kefyr*, *Candida krusei*, and *Candida glabrata*, but its concentration and biofilm formation are the highest for *C. albicans* [29].

In *Candida*, several proteins’ and genes’ expressions have been shown to be affected by Far. Cao et al. reported that, in the presence of Far, *TUP1* (general transcriptional corepressor 1 gene), *CRK1* (serine/threonine-protein kinase 1 gene), and *PDE2* (phosphodiesterase 2 gene related to hyphal formation), *FCR1* (Fluconazole resistance 1 gene) and *PDR16* (phosphatidylinositol transfer gene, related to drug resistance), *CHT2* and *CHT3* (chitinase 2 and 3 genes, related to cell wall maintenance), *FTR2* (formylmethanofuran-tetrahydromethanopterin formyltransferase, for iron transport), and *HSP70*, *HSP90*, *HSP104*, *CaMSI3*, and *SSA2* (encoding heat shock proteins) are upregulated. *CSH1* (cell surface hydrophobicity) has a downregulation response in the presence of Far [30]. Similarly, Far has been demonstrated to suppress the resistance of *C. albicans* biofilms to antifungals by regulating the expression of *CYR1* (adenylate cyclase gene, involved in regulation of filamentation, phenotypic switching, and mating) and *PDE2* (moderates signaling by cyclic adenosine monophosphate-cAMP; required for virulence). *PDE2* regulation was subordinated to *CYR1* regulation [31]. Far has also been proven to downregulate secreted aspartyl proteinases (Saps) 2, 4, 5, and 6 mRNA expression, which indicates that this QSM modules *Candida* morphogenesis [32]. The same work indicates that, in *C. albicans*, Far inhibits hyphal growth by controlling the cAMP signaling pathway [32]. Similarly, Far is linked to the inhibition of the translation to constrain growth and filamentation in yeasts (*C. albicans* and *S. cerevisiae*), targeting a singular step [27]. Polke and colleagues indicated eed1Δ/Δ (EED1—crucial for hyphal extension and maintenance) as the first Far hypersensitive mutant of *C. albicans*. This mutant strain was described as excreting 10 times more Far, and, although being able to form hyphae, it cannot preserve these forms [33]. Instead, the conservation of hyphal growth is thought to raise the Far reaction threshold. Curiously, dpp1p, dpp2p, and dpp3p (non-specific dipeptide pyrophosphatases/permeases responsible for Far synthesis) do not explain differences in Far levels involving the participation of supplementary factors (e.g., scaffolding molecules) [34]. The inhibition of hyphal initiation has been shown to be mostly performed by blocking the protein degradation of Nrg1 (a repressor of hyphal development), related to Far, which is connected to the activation of the cAMP-PKA (protein kinase A) pathway and, thus, to the initial steps of hyphal growth [35]. This compound induces reactive oxygen species (ROS) production and increases resistance to oxidative stress [36]. However, an influence of Far on *C. albicans* yeasts is dependent on used concentrations. While higher concentrations (200–300 μM) are stressful for yeasts, lower concentrations (about 40 μM) protect them from stress [23,37,38]. Moreover, in a recent work, a nanogel with alginate and chitosan polymers containing 300 µM of Far was used as a nanocarrier for pharmaceutical application of this QSM. The results indicated that *C. albicans* expression of *HWP1* (hyphal wall precursor gene) and *SAP6* (secreted aspartyl) genes were pointedly reduced, after the application of this novel nanogel with Far against *C. albicans* [39]. In addition, the study of the effects of Far on *C. dubliniensis* biofilm indicated a synergy between Far and fluconazole in resistant strains. This led to a reversal of fluconazole resistance, which is indeed a crucial result that suggests a possible application of Far as an adjuvant therapeutic agent [40].

Indeed, filamentation and QS are vital factors in *C. albicans* biofilm development. Ramage et al. revealed that the effect of Far is dependent on its concentration in the early adherence period. The authors signposted that a preincubation Far entirely inhibited biofilm formation, as evidenced by a morphogenetic autoregulatory effect exerted by this compound. The expression of *HWP1* (which encodes a hypha-specific wall protein) diminished in biofilms treated with Far, which validated a possible use of Far as a new drug [6]. In another report, Far has also been revealed to change the sensitivity of *C. albicans* cells to oxidants. In fact, a *Candida*-conditioned growth medium induced the expression of *CAT1* (peroxisomal catalase 1 gene), *SOD1*, *SOD2* (superoxide dismutase genes), and the results indicated that this protection might be controlled by the transcriptional regulation of antioxidant-encoding genes, and, henceforth, linked to the oxidative stress response in *C. albicans* [21]. Notably, the phenotypic switching of *Candida* plays an important role in the development of infection. As previously mentioned, Far inhibits transition from the yeast morphotype to hyphal cells [20]; however, it cannot completely abolish hyphal development, denoting that additional unknown inhibitory molecules with similar function must exist [4]. Nonetheless, the mechanism underlying this ability is still completely unclear. Regarding the sterol synthesis pathway, which involves the synthesis of Far, *ERG25* (methylsterol monooxygenase) and *ERG4* (delta 24(24(1))-sterol reductase) were both shown to be downregulated in the Far-exposed group. It was also concluded that exogenous Far has an evident, but a non-deterministic effect on the synthesis of ergosterol [41]. Likewise, externally added Far also triggers morphological features characteristic of apoptosis, mediated by ROS in *Aspergillus nidulans* and *Fusarium graminearum*, and appears to protect *Candida* from oxidative stress. Although Far induces accumulation of intracellular ROS in *Candida*, this does not appear to be a mechanism of oxidative stress protection/resistance, since α-tocopherol and ascorbic acid (antioxidants) failed the attenuation of Far-mediated ROS [4,21]. Singkum et al. confirmed that tryptophol can trigger apoptosis and reduce the virulence of *C. albicans in vivo*. Both Far and tryptophol inhibit *C. albicans* germ tube formation, and the expression levels of the apoptosis genes increases, while the expression level of the anti-apoptosis gene reduces [28]. Recently, it has been validated that a robust hyphal development involves downregulation of two transcriptional repressors, Nrg1 (nucleic acid binding protein) and Sfl1 (suppressor protein for flocculation), and that acidic pH or cationic stress can inhibit hyphal formation, via stress-responsive kinases and Sfl1 [42]. Also, and for the first time, it was indicated that only Far (but not farnesoic acid or Tyr) is able to activate the extracellular traps’ (neutrophil extracellular traps, netosis (NETs)) formation, through selective inhibitors of the NET signaling pathway. Mac-1 (macrophage antigen-1) and TLR2 (toll-like 2) receptors were found to be responsible for Far identification and activation of the ROS-dependent netosis pathway [43]. Another important point is the cell wall remodeling in *C. albicans.* This mechanism is known to help escaping or hyperactivating the host’s innate immune responses, leading to disease. Re-masking of β-glucan is equally promoted by Far, while chitin re-masking is controlled via other small, heat-stable, non-proteinaceous secreted molecule(s). A recent study indicates that, by exposing *C. albicans* to an acidic environment (such as it is in the stomach or vagina), detection of the yeast by macrophages rises. Nonetheless, this pH effect is transitory, as *C. albicans* can re-mask these epitopes (glucan and chitin) [44].

Lastly, many studies of single or mixed-species biofilms observed effects of Far (i) produced by one species (mainly *Candida*) and affecting the presence of another one in this community or (ii) exogenously added. Results from a Kong et al.’s work demonstrated that, in the presence of externally supplemented Far or Far secreted by *C. albicans* in biofilm, *Staphylococcus aureus* exhibited significantly enhanced tolerance to antimicrobials [45]. As a further matter, the crucial role of *C. albicans*-secreted Far in the modulation of *S. aureus’* response to antimicrobials in mixed biofilms has also been demonstrated. *S. aureus* Far-induced transcriptional modulations of key regulatory networks can modulate the pathogenesis of mixed *C. albicans–S. aureus* co-infections. The sensitized *S. aureus* phenotype exhibited dramatic loss of the typical pigment—staphyloxanthin, an important virulence factor [46]. Similarly, Far has been shown to be active against *Staphylococcus epidermidis*, the biofilm biomass reduction was not a result of cell killing but of biofilm detachment by exogenously added Far [47]. Research by Cugini et al. examined interactions in another dual-species biofilm, when *C. albicans*-produced Far stimulates *Pseudomonas aeruginosa* quinolone signal production in LasR-defective (a *quorum-sensing* signal receptor) *P. aeruginosa* strains, because *lasR* mutants lacked the master QS system regulator [48]. Another interesting finding revealed that codelivery of Far and ciprofloxacin seems to be a promising approach to battle antibiotic-resistant *P. aeruginosa* biofilms by enhancing biofilm killing at significantly lower antibiotic doses [49]. In the case of *Paracoccidioides brasiliensis* dimorphism, it was described that adding Far retarded the germ tube formation, probably associated to cytoplasmic degeneration [26].

## 3. Tyrosol

**Figure 4:**
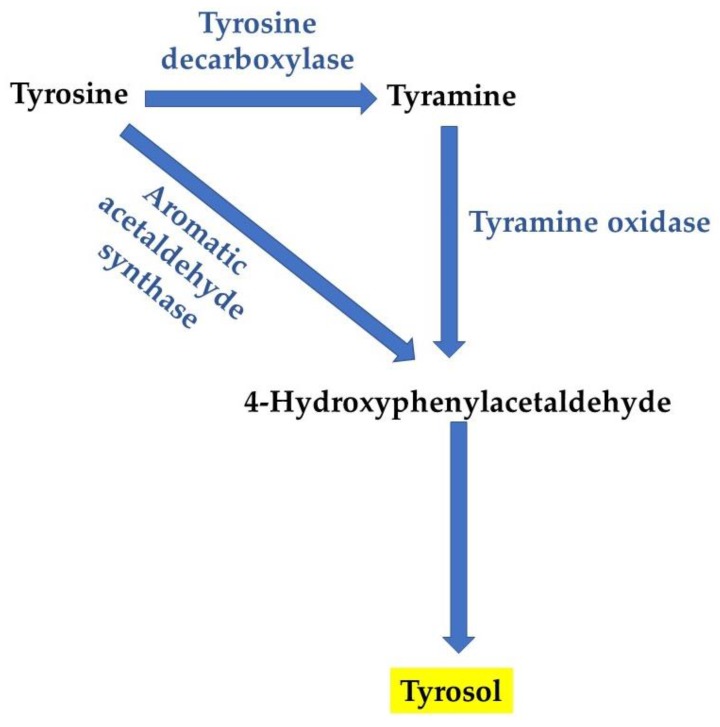
Scheme of Tyr biosynthesis, according to Reference [50].

Tyr, (2-(4-hydroxyphenyl)-ethanol (Figure 1), belongs to a group of phenolic compounds called phenylethanoids [50]. Together with hydroxytyrosol, they are the main phenolic compounds found in the virgin olive oil [51]. For example, in plants, Tyr synthesis is achieved from tyrosine, with two possible biosynthesis pathways (Figure 4) [50]. In the first proposed pathway, tyrosine is converted into tyramine by tyrosine decarboxylase. Subsequent oxidation and reduction of tyramine result in the formation of Tyr [52]. However, growing evidence indicates that Tyr is synthesized via tyramine, as tyrosine decarboxylase was identified in *Rhodiola sachalinensis* [53,54]. As a matter of fact, Tyr is a powerful antioxidant compound, possibly more due to intracellular accumulation than to the antioxidant activity itself, which is weak compared with other molecules. Antioxidant activity is induced by scavenging ROS and nitrogen species that are related to human disease [55]. On the other side, its antibacterial activity is exerted by binding and inhibiting bacterial ATP synthase [56].

*C. albicans*’ yields of Tyr and other aromatic alcohols (e.g., phenethyl alcohol, tryptophol) are defined by growth conditions, comprising oxygen levels, aromatic amino acids and ammonium salts availability, and pH. Tyr also seems to be controlled in *S. cerevisiae*, equally dependent on the cell density [15]. In diluted *Candida* cultures, Tyr worked as an active compound released into the medium continuously during growth, accelerating the formation of germ tubes. Tyr shortened the lag phase and accelerated the morphological conversion of *Candida* yeast-form cells to filamentous protrusion [11]. Ghosh et al. disclosed that, in *C. albicans*, the production of Tyr varies just by adding tyrosine or ammonium salts in the growth medium. The transcription regulator Aro80p was shown to also be responsible for the aromatic alcohol production, such as Tyr. The expressions of genes such as *ARO8*, *ARO9*, and *ARO10* (aromatic amino-acid genes) are equally pH-dependent, specifically: *ARO8* and *ARO9*—alkaline upregulated, and *ARO10*—alkaline downregulated. Moreover, the alkaline-dependent alteration in *ARO8* expression is Rim101-independent (a pH-response transcription factor), and *ARO9* expression is Rim101-dependent [57]. Tyr secretion and dpp3 protein are linked and can modulate the secretion of Tyr and phenethyl alcohol (signaling molecules in *Candida*) [58]. Also, a study concluded that the stimulation of a quicker transition from yeast form to hyphal cells, under favorable conditions, is also influenced by Tyr [11]. A similar report with mutants (*cappz1* and *hgc*, fungus-specific protein phosphatase Z1—*CaPPZ1*, and the hypha-specific cyclin—*HGC1*) revealed that Tyr is responsible for a firm adherence and confirmed the faster yeast-to-hypha transition. Importantly, this work concluded that yeasts’ attachment, yeast-to-hyphal transition, and hyphal growth rate are strictly related processes [59]. In fact, when diluted into fresh minimal medium, *C. albicans* growth has a considerable lag effect. This is reduced by continuously adding Tyr through a conditioned medium from a high-density culture. In permissive conditions for germ tube formation, Tyr stimulates their formation. On the contrary, as the germ tube formation is constrained by Far, the process is thus assumed to be under complex control by environmental states [11].

Indeed, Tyr plays a key role in fungal morphogenesis and biofilm development, and a link between Tyr assembly and biomass for both planktonic and biofilm cells has been determined. During biofilm development, Tyr can stimulate hypha production throughout initial stages (1–6 h), acting as a QSM for both cells, with a more powerful action in the early and intermediate periods of biofilm formation [60]. This molecule also has a remarkable antifungal effect at supraphysiological concentrations, but the background remains unknown, especially in the case of non-*Candida albicans Candida* species, such as *C. glabrata* or *C. parapsilosis.* Interestingly, the interaction between fluconazole and Tyr has been studied and concluded as antagonistic. Tyr exposure was revealed to enhance the oxidative stress response and raise the efflux pumps’ gene expressions, while inhibiting several virulence-related genes, growth, and ribosome biogenesis. Additionally, cells’ metabolism was altered for fermentation mechanisms, such as the ones involving ethanol and glycolysis. Still, in this report, adherence in the beginning was not considerably induced in the presence of Tyr [61]. Not less important, in a recent study that evaluated mixed *P. aeruginosa–C. albicans* biofilms, Tyr had an antibacterial activity, toughly inhibiting the production of hemolysin and protease in *P. aeruginosa*, while Far inhibited hemolysin production [62]. Table 1 summarizes these activities for Far and Tyr.

## 4. Biofilm Formation: Role of Farnesol and Tyrosol

**Figure 5:**
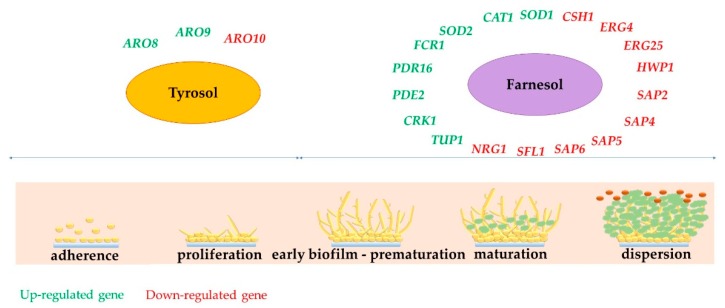
Genes participating in biofilm formation impacted by Far and Tyr.

Understanding the mechanisms of action of Far and Tyr can lead to the development of new antifungal compounds, targeting *Candida* biofilms, possibly leading biofilms to regain more sensitity to antibiotics. Despite the many available findings about pathways affected by Far, less is known about Tyr effects. Hence, we cannot exactly conclude, with a deeper knowledge, which genes’ expression in *Candida* biofilm are directly impacted by these QSM. A suggestion is presented in Figure 5, according to the main reports, related to the mechanism of action of Far and Tyr on morphological changes.

Biofilm development and behavior of cells in the presence of QSM is concentration-dependent, even if molecules are directly synthesized or exogenously added. A simultaneous addition of Tyr and Far at different concentrations have indicated that Far was dominant and 48 h matured biofilms mainly presented spherical cells (not hypha/not mycelial form) [60]. The authors also tested the ability of supernatants to influence on germ tube formation (planktonic cells). In this case, Tyr activity exceeded Far after 14 h, but not after 24 h. As such, exogenous Tyr was able to stimulate hypha production during the early stages (1 to 6 h) and intermediate stages of biofilm development before some cells are already committed to hyphal growth. It was confirmed that Tyr acts as a QSM for biofilms as well as for planktonic cells [60]. On the other hand, Far activity increased significantly during the later stages (48 to 72 h) of biofilm development [60], meaning that, in mature biofilms, Far activity and concentration surpass Tyr and possibly have a critical role on the release of yeast cells for biofilm dispersal, which was also suggested before [3,6].

Dižová et al. revealed that Far inhibits biofilm formation on *C. albicans*. Indeed, in combination with fluconazole, Far induced an upregulation of *ERG9* on *C. albicans* biofilms. Yet, the same study revealed that the highest concentration of Far (200 μM) was more effective [63]. In a previous study, Far inhibited hyphal growth and the expression of genes was necessary for a robust biofilm formation. Several steps of biofilm development are influenced by Far. Among them, the architecture of mature biofilms, the adherence of cells to the substratum, and the biofilm cells’ dispersion, are the most relevant [64]. *Candida auris* is a severe global health threat due to a key multidrug-resistant pattern. This yeast can form biofilm, exhibiting decreased susceptibility to echinocandins, which is associated with poorer clinical outcomes. As a QSM, Far had a prominent effect with echinocandins against *C. auris* biofilms [65]. Importantly, cells constrain the cell number of intense biofilms, by liberating self-inhibitory compounds. Actually, Tyr, 2-phenylethanol, and Far, were identified in *C. tropicalis* cultures. Far amplified the inhibition exerted by natamycin, which reduced the biofilm formation, growth and expansion, from juice on stainless steel surfaces. This has highlighted the possibility of using Far in the food industry (or other QSM) [66]. In biofilms treated with higher concentrations of Far, the addition of Tyr resulted in biofilms containing the majority of cells in the yeast form, Tyr could not counteract the effects of higher concentrations of Far [3] and presumably, the effects of Far predominate [11].

Regarding Tyr, reports have also been published. As an inducer of biofilm formation, Tyr has been recognized as endorsing the biofilm-forming ability of *C. auris*, to grow as yeast or pseudohyphae [12]. Importantly, regarding medical devices, intrauterine contraceptives were evaluated. These devices are a compact surface for microbial attachment and the development of biofilms. Using 80 μM Tyr combined with 4 mg/L of amphotericin B, approximately 90% of *Candida krusei* and *C. tropicalis* biofilms were reduced, showing them to be suitable for this effect [67]. Regarding oral health, studies have different results. A combination of Tyr and Far has been explored for oral *Candida* isolates in both planktonic and biofilm cells. This combination was beneficial for specific parameters against oral *Candida*, but synergy was merely noticed for *C. glabrata*. These results indicate that a combination of Tyr and Far can contribute, to a certain point, to the development of oral care products to combat *Candida* infections [68]. Another similar study with *C. albicans* strains, isolated from dentures, proved a particular anti-biofilm activity, sovereign fungicidal or fungistatic effect, of Far and Tyr [69]. On the contrary, a different report suggested that the single use of Tyr was not capable to pointedly decrease hydrolytic enzymes and acid production on oral *Candida* and *Streptococcus mutans*. Tyr showed a limited efficacy against these single and mixed-species oral biofilms [70]. Ultimately, the combination of poly(vinyl alcohol)-coated silver nanoparticles and Far proved to have antimicrobial and anti-adhesion activities, which indicate the possibility of using this combination as a co-adjuvant in endodontic treatments, or an alternative assisting method for root canal disinfection to prevent biofilm formation [71]. In another study, the impact of exogenous Tyr was investigated to be synergic to antifungals targeting cellular ergosterol. Interestingly, mature biofilms were susceptible to Tyr alone or in combination with amphotericin, but Tyr with azoles enhanced biofilm growth [72]. Also, a combination of Tyr and chlorhexidine gluconate effectively reduced only the number of *C. albicans* hyphae, but these agents were ineffective against tested *C. albicans*, *C. glabrata*, and *S. mutans* biofilms [73]. The findings of Kovács et al. describe *in vitro* activity of caspofungin and micafungin against *C. parapsilosis* biofilms in the presence of Tyr, when metabolic activity reduction and cell damage was detected [74]. In fact, there is lack of published information related to Tyr and differential gene expression in Tyr-treated biofilms has not been reported. However, mutants of *C. albicans* with defined defects in the Efg1 (enhanced filamentous growth protein 1), the Cph1 (transcription factor CPH1), or both morphogenetic signaling pathways also produced Tyr in a density-dependent fashion and at levels similar to that of the wild-type strain [60].

Several works have described the addition of Far to bacterial biofilms and have indicated a promising synergic effect with common antibiotic therapy in both fungi and bacteria. For example, the sensitization of methicillin-resistant *S. aureus* strains and the synergistic effect of Far and gentamicin has supported the application of this QSM as an adjuvant in anti-biofilm therapy [75]. After being exogenously administrated or secreted by *C. albicans* Far, *S. aureus* biofilms demonstrated significant tolerance to antibiotics [45]. On the contrary, another research showed that in mixed *C. albicans* and *S. mutans* biofilm, bacterial growth was not affected after the addition of 200 μM of Far [76]. Also, Far was tested to be a molecule with the capability to break the extracellular matrix of *Fusarium keratoplasticum*, demonstrating its anti-biofilm activity, causing the destruction of hyphae and preventing the adherence of conidia, filamentation, and biofilm formation [77].

## 5. Future Remarks

The ability of microbes to communicate as an entire group is beneficial during colonization of host niche, biofilm development, adaptation processes, or defense against competitors. The secretion of signaling molecules is increased and represents an interesting communication QS system. These molecules are released by both fungal and bacterial cells and have an autoinducing function. When microbial density reaches threshold, cell´s receptors are activated and their signal affects genes’ expression, resulting in coordinated community feedback. The best described QS process is for *C. albicans*, for which two signal molecules (Far and Tyr) were discovered that manage the transition between spherical cells and hyphal form, among other relevant processes that are still to be fully understood.

As a matter of fact, the anti-biofilm effect of Far is clearly shown when this compound is added to various stages of biofilm formation, but an opposite role is attributed to Tyr in this process, which should be better explored. Importantly, both molecules have an antifungal effect that requests further research, alone or in combination with other compounds (perfectly identified or still under study), in order to promote innovative therapies to fight *Candida* infections. Indeed, unveiling the mechanisms of action can be helpful to explore and design new antifungal drugs, which are potentially more effective and less toxic for the treatment of *Candida* infections. However, there are still several questions to be answered and mechanisms to be defined and comprehended. This is the case for the development of Far and Tyr interactions with other microbes and host cells, the mode of these two QSMs’ transport across the cell, and all the cell receptors involved for each of the molecules’ responses, but also factors such as the possibility of the existence of new QSMs and new pathways.

## Figures and Tables

**Table 1:** General Far and Tyr roles in *Candida.*

*Quorum-sensing* Molecule	Activity	Reference(s)
Farnesol	Inhibition of hypha formation, filamentation, and biofilm formation/development	[6,13,14,27,28]
	Regulation of drug efflux and apoptosis	[4,5,14,28]
	Anti-cancer/anti-tumor, anti-inflammatory, anti-allergic, and anti-obesity	[14]
	Fungicidal, antimicrobial	[14]
	Inhibition of the transition from the oval/spherical cell morphotype to hyphal cells	[4,20]
Tyrosol	Antioxidant	[55,56]
	Cells’ stimulation of a quicker transition from oval/spherical cell to hyphal form	[11,59]
	Induction of germ tube formation	[11]
	Stimulation of firm adherence of the cells to surfaces	[59]
	Initiation of biofilm formation	[60]
	Antifungal	[60]
